# The TRAPP Subunit Trs130p Interacts with the GAP Gyp6p to Mediate Ypt6p Dynamics at the Late Golgi

**DOI:** 10.3389/fcell.2016.00048

**Published:** 2016-05-24

**Authors:** Stephanie Brunet, Djenann Saint-Dic, Miroslav P. Milev, Tommy Nilsson, Michael Sacher

**Affiliations:** ^1^Department of Biology, Concordia UniversityMontreal, QC, Canada; ^2^Department of Medicine, McGill UniversityMontreal, QC, Canada; ^3^Department of Anatomy and Cell Biology, McGill UniversityMontreal, QC, Canada

**Keywords:** Gyp6, TRAPP, Ypt6, GEF, GAP, Ypt31, Ypt32

## Abstract

Small GTPases of the Rab superfamily participate in virtually all vesicle-mediated trafficking events. Cycling between an active GTP-bound form and an inactive GDP-bound form is accomplished in conjunction with guanine nucleotide exchange factors (GEFs) and GTPase activating proteins (GAPs), respectively. Rab cascades have been described in which an effector of an activated Rab is a GEF for a downstream Rab, thus ensuring activation of a pathway in an ordered fashion. Much less is known concerning crosstalk between GEFs and GAPs although regulation between these factors could also contribute to the overall physiology of a cell. Here we demonstrate that a subunit of the TRAPP II multisubunit tethering factor, a Rab GEF, participates in the recruitment of Gyp6p, a GAP for the GTPase Ypt6p, to Golgi membranes. The extreme carboxy-terminal portion of the TRAPP II subunit Trs130p is required for the interaction between TRAPP II and Gyp6p. We further demonstrate that TRAPP II mutants, but not a TRAPP III mutant, display a defect in Gyp6p interaction. A consequence of this defective interaction is the enhanced localization of Ypt6p at late Golgi membranes. Although a *ypt31/32* mutant also resulted in an enhanced localization of Gyp6p at the late Golgi, the effect was not as dramatic as that seen for TRAPP II mutants, nor was Ypt31/32 detected in the same TRAPP II purification that detected Gyp6p. We propose that the interaction between TRAPP II and Gyp6p represents a parallel mechanism in addition to that mediated by Ypt31/32 for the recruitment of a GAP to the appropriate membrane, and is a novel example of crosstalk between a Rab GAP and GEF.

## Introduction

Vesicles containing lipids and protein cargo are transported throughout the cell in a highly regulated manner (Palade, 1975; Novick et al., 1980; Balch et al., 1994). Several factors cooperate to ensure that vesicles arrive at the correct compartment, among them Rab proteins and tethering factors (Bonifacino and Glick, 2004; Cai et al., 2007). Rab proteins are small GTPases located throughout the endomembrane system and act as molecular switches, transitioning from a GTP-bound (“on”) to a GDP-bound (“off”) state upon hydrolysis of GTP (Zerial and McBride, 2001; Seabra and Wasmeier, 2004; Yu and Hughson, 2010). Due to their inherently low ability to exchange GDP for GTP and subsequently, hydrolyze GTP, Rab proteins require guanine nucleotide exchange factors (GEFs) and guanine nucleotide activating proteins (GAPs) to promote exchange of GDP for GTP and hydrolysis of GTP to GDP, respectively (Bourne et al., 1990; Bos et al., 2007; Barr and Lambright, 2010). The coordinated recruitment of Rabs and their respective GEFs and GAPs to specific intracellular locations regulates the initiation and termination of membrane trafficking pathways. Rab GEF and Rab GAP cascades have been proposed to proceed in opposite directions and mediate sequential Rab activation and inactivation, respectively (Nottingham and Pfeffer, 2009). Each Rab within these cascades recruits the GEF for the next Rab and the GAP for the previous Rab in the pathway, providing directionality and limiting the localization of Rabs to specific compartments. Examples of these cascades have been described in secretory and endosomal trafficking pathways (Ortiz et al., 2002; Wang and Ferro-Novick, 2002; Rivera-Molina and Novick, 2009; Suda et al., 2013). In addition to Rabs, several other factors have been identified that contribute to the correct localization of Rab GEFs, including vesicle coat proteins and SNAREs (soluble *N*-ethylmaleimide-sensitive factor attachment protein receptor) (Barr, 2013). In contrast, aside from their interaction with Rabs, very few factors have been identified that mediate the recruitment of Rab GAPs to specific compartments. Rab GEFs are interesting candidates to promote Rab GAP recruitment to the correct location as they are diverse in their structure and composition and are directly involved in Rab activation. A GEF-GAP interaction could also create cross-talk between opposing Rab GAP and Rab GEF cascades, providing an additional layer of control to ensure that Rabs are turned “on” and “off” in a coordinated manner.

Ypt6p is a non-essential Rab in yeast that is implicated in several trafficking pathways including post-Golgi trafficking, endosome to Golgi transport, retrograde transport through the Golgi, endoplasmic reticulum (ER)-to-early Golgi transport, early Golgi-to-ER transport, and autophagy (Bensen et al., 2001; Luo and Gallwitz, 2003; Liu et al., 2006; Ohashi and Munro, 2010; Benjamin et al., 2011). Ypt6p is activated by the heterodimeric Ric1p/Rgp1p complex, and inactivated by the GAP protein Gyp6p (Strom et al., 1993; Siniossoglou et al., 2000; Will and Gallwitz, 2001). The GAPs Gyp2p and Gyp3p have also exhibited activity toward Ypt6p *in vitro* (Albert and Gallwitz, 1999). Like Ypt6p, Ric1p, Rgp1p, and Gyp6p are all not essential for cell viability (Strom et al., 1993; Siniossoglou et al., 2000).

Transport protein particle (TRAPP) represents a family of three related protein complexes in yeast called TRAPP I, II, and III (Kim et al., 2016b). All three complexes have been suggested to act as a GEF for the Ypt1p Rab GTPase, and all three function in distinct trafficking processes including ER-to-Golgi (TRAPP I), anterograde and retrograde traffic at the late Golgi (TRAPP II) and autophagy (TRAPP III). A Rab GAP cascade was recently described involving the recruitment of Gyp6p by active Ypt31/Ypt32p, a redundant Rab pair localized to the late Golgi, to promote the dissociation of Ypt6p from these membranes (Suda et al., 2013). The multisubunit tethering complex TRAPP II acts as a GEF for the early Golgi Rab Ypt1p as well as a putative GEF for the Ypt31/32p Rab pair (Jones et al., 2000; Wang et al., 2000; Morozova et al., 2006; Cai et al., 2008; Lynch-Day et al., 2010; Yip et al., 2010). There is overlap between TRAPP II- and Ypt6p-regulated membrane trafficking pathways as TRAPP II acts in post-Golgi trafficking from the late Golgi to the plasma membrane and early endosome to late Golgi transport (Sacher et al., 2000, 2001; Cai et al., 2005; Montpetit and Conibear, 2009; Yip et al., 2010; Choi et al., 2011). Here we present evidence that the TRAPP II complex promotes the dissociation of Ypt6p from the late Golgi through the recruitment of the Ypt6p GAP Gyp6p. Destabilization of the TRAPP II complex disrupts its association with Gyp6p and causes Ypt6p to become enriched at the late Golgi, a phenotype also observed in a *gyp6*Δ mutant. The interaction between TRAPP II, a Ypt1p and putative Ypt31/32p GEF, and the Rab GAP Gyp6p, is a novel mechanism for the recruitment of a GAP to the appropriate membrane and may also be an example of cross talk between a Rab GAP and Rab GEF cascade.

## Materials and methods

### Strain construction

A list of all plasmids and yeast strains used in this study is provided in Tables 1, 2, respectively. In wild type and mutant strains, *GYP6* was tagged at the carboxy terminus with a triple hemagglutinin (HA) epitope by genomic insertion of a cassette amplified from pFA6a-3HA-His3MX6 or pFA6a-3HA-TRP1MX6 (Longtine et al., 1998). Insertion at the correct location was verified by PCR and Western blot analysis.

**Table 1:** **Plasmids used in this study**.

		**Source**
MSB238	*pRS425*	
MSB247	*pRS425-BET5*	
MSB248	*pRS425-TRS20*	
MSB281	*pRS425-TRS23*	
MSB290	*pRS425-BET3*	
MSB271	*pRS425-TRS31*	
MSB250	*pRS425-TRS33*	
MSB473	*pRS425-TCA17*	
MSB291	*pRS425-TRS65*	
MSB284	*pRS425-TRS85*	
MSB285	*pRS425-TRS120*	
MSB474	*pRS425-TRS130*	
MSB1063	*pRS315-GFP-YPT6*	Ruth Collins laboratory
MSB1319	*pRS316-ADH1pr-SEC7-mRFP*	Akihiko Nakano laboratory (Suda et al., 2013)
MSB1321	*pRS306-ADH1pr-mRuby-SED5*	Akihiko Nakano laboratory (Suda et al., 2013)

**Table 2:** **Yeast strains used in this study**.

MSY20	*MATa his3-11 leu2-3,112 ura3-1 ade2-1 trp1Δ can100*
MSY49	*MATa his3Δ1 ura3Δ0 leu2Δ0 met15Δ0 TRS120-TAP::HIS3MX6*
MSY135	*MATα his3Δ1 leu2Δ0 lys2Δ0 ura3Δ0*
MSY366	*MATa trp1-901 leu2-3, 112 ura3-52 his3-200 gal4*Δ *gal80*Δ *LYS2::GAL1_*UAS*_-GAL1_*TATA*_-HIS3 GAL2_*UAS*_-GAL2_*TATA*_-ADE2 URA3::MEL1_*UAS*_-MEL1_*TATA*_-lacZ pGADT7-BET5*
MSY367	*MATa trp1-901 leu2-3, 112 ura3-52 his3-200 gal4*Δ *gal80*Δ *LYS2::GAL1_*UAS*_-GAL1_*TATA*_-HIS3 GAL2_*UAS*_-GAL2_*TATA*_-ADE2 URA3::MEL1_*UAS*_-MEL1_*TATA*_-lacZ pGADT7-TRS20*
MSY368	*MATa trp1-901 leu2-3, 112 ura3-52 his3-200 gal4*Δ *gal80*Δ *LYS2::GAL1_*UAS*_-GAL1_*TATA*_-HIS3 GAL2_*UAS*_-GAL2_*TATA*_-ADE2 URA3::MEL1_*UAS*_-MEL1_*TATA*_-lacZ pGADT7-BET3*
MSY369	*MATa trp1-901 leu2-3, 112 ura3-52 his3-200 gal4*Δ *gal80*Δ *LYS2::GAL1_*UAS*_-GAL1_*TATA*_-HIS3 GAL2_*UAS*_-GAL2_*TATA*_-ADE2 URA3::MEL1_*UAS*_-MEL1_*TATA*_-lacZ pGADT7-TRS23*
MSY370	*MATa trp1-901 leu2-3, 112 ura3-52 his3-200 gal4*Δ *gal80*Δ *LYS2::GAL1_*UAS*_-GAL1_*TATA*_-HIS3 GAL2_*UAS*_-GAL2_*TATA*_-ADE2 URA3::MEL1_*UAS*_-MEL1_*TATA*_-lacZ pGADT7-TRS31*
MSY371	*MATa trp1-901 leu2-3, 112 ura3-52 his3-200 gal4*Δ *gal80*Δ *LYS2::GAL1_*UAS*_-GAL1_*TATA*_-HIS3 GAL2_*UAS*_-GAL2_*TATA*_-ADE2 URA3::MEL1_*UAS*_-MEL1_*TATA*_-lacZ pGADT7-TRS33*
MSY372	*MATa trp1-901 leu2-3, 112 ura3-52 his3-200 gal4*Δ *gal80*Δ *LYS2::GAL1_*UAS*_-GAL1_*TATA*_-HIS3 GAL2_*UAS*_-GAL2_*TATA*_-ADE2 URA3::MEL1_*UAS*_-MEL1_*TATA*_-lacZ pGADT7-TRS65*
MSY373	*MATa trp1-901 leu2-3, 112 ura3-52 his3-200 gal4*Δ *gal80*Δ *LYS2::GAL1_*UAS*_-GAL1_*TATA*_-HIS3 GAL2_*UAS*_-GAL2_*TATA*_-ADE2 URA3::MEL1_*UAS*_-MEL1_*TATA*_-lacZ pGADT7-TRS85*
MSY374	*MATa trp1-901 leu2-3, 112 ura3-52 his3-200 gal4*Δ *gal80*Δ *LYS2::GAL1_*UAS*_-GAL1_*TATA*_-HIS3 GAL2_*UAS*_-GAL2_*TATA*_-ADE2 URA3::MEL1_*UAS*_-MEL1_*TATA*_-lacZ pGADT7-TRS120*
MSY375	*MATa trp1-901 leu2-3, 112 ura3-52 his3-200 gal4*Δ *gal80*Δ *LYS2::GAL1_*UAS*_-GAL1_*TATA*_-HIS3 GAL2_*UAS*_-GAL2_*TATA*_-ADE2 URA3::MEL1_*UAS*_-MEL1_*TATA*_-lacZ pGADT7-TRS130*
MSY376	*MATa trp1-901 leu2-3, 112 ura3-52 his3-200 gal4*Δ *gal80*Δ *LYS2::GAL1_*UAS*_-GAL1_*TATA*_-HIS3 GAL2_*UAS*_-GAL2_*TATA*_-ADE2 URA3::MEL1_*UAS*_-MEL1_*TATA*_-lacZ pGADT7-TCA17*
MSY377	*MATa trp1-901 leu2-3, 112 ura3-52 his3-200 gal4*Δ *gal80*Δ *LYS2::GAL1_*UAS*_-GAL1_*TATA*_-HIS3 GAL2_*UAS*_-GAL2_*TATA*_-ADE2 URA3::MEL1_*UAS*_-MEL1_*TATA*_-lacZ pGADT7*
MSY538	*MATα ura3-52 his3-200 ade2-101 trp1-901 leu2-3, 112 met- gal4Δ gal80Δ URA3::GAL1_*UAS*_-GAL1_*TATA*_-lacZ pGBKT7(gateway modified)-GYP6*
MSY540	*MATα his3Δ1 leu2Δ0 lys2Δ0 ura3Δ0 ypt6Δ::KanMX*
MSY609	*MATα his3Δ1 leu2Δ0 lys2Δ0 ura3Δ0 ric1Δ::KanMX*
MSY611	*MATα his3Δ1 leu2Δ0 lys2Δ0 ura3Δ0 rgp1Δ::KanMX*
MSY646	*MATα his3Δ1 leu2Δ0 lys2Δ0 ura3Δ0 GYP6-3xHA::HIS3 BY4742*.
MSY647	*MATα his3Δ1 leu2Δ0 lys2Δ0 ura3Δ0 YEL048cΔ::KanMX GYP6-3xHA::HIS3*
MSY658	*MATα his3Δ1 leu2Δ0 lys2Δ0 ura3Δ0 ric1Δ::KanMX GYP6-3xHA::HIS3*
MSY659	*MATα his3Δ1 leu2Δ0 lys2Δ0 ura3Δ0 ypt6Δ::KanMX GYP6-3xHA::HIS3*
MSY660	*MATα his3Δ1 leu2Δ0 lys2Δ0 ura3Δ0 rgp1Δ::KanMX GYP6-3xHA::HIS3*
MSY661 MSY677	*MATa his3-11 leu2-3,112 ura3-1 ade2-1 trp1Δ can100 trs130Δ50^*ts*^::TRP1 MATα his3Δ1 leu2Δ0 lys2Δ0 ura3Δ0 trs65Δ::KanMX GYP6-3xHA::HIS3*
MSY678	*MATα his3Δ1 leu2Δ0 lys2Δ0 ura3Δ0 trs85Δ::KanMX GYP6-3xHA::HIS3*
MSY679	*MATα his3Δ1 leu2Δ0 lys2Δ0 ura3Δ0 trs33Δ::KanMX GYP6-3xHA::HIS3*
MSY680	*MATα his3Δ1 leu2Δ0 lys2Δ0 ura3Δ0 vps51Δ::KanMX GYP6-3xHA::HIS3*
MSY771	*MATα his3Δ1 leu2Δ0 lys2Δ0 ura3Δ0 pRS315-GFP-YPT6*
MSY772	*MATα his3Δ1 leu2Δ0 lys2Δ0 ura3Δ0 trs65Δ::KanMX pRS315-GFP-YPT6*
MSY775	*MATα his3Δ1 leu2Δ0 lys2Δ0 ura3Δ0 ric1Δ::KanMX pRS315-GFP-YPT6*
MSY776	*MATα his3Δ1 leu2Δ0 lys2Δ0 ura3Δ0 gyp6Δ::KanMX pRS315-GFP-YPT6*
MSY777	*MATα his3Δ1 leu2Δ0 lys2Δ0 ura3Δ0 pRS315-GFP-Ypt6 pRS316-ADH1pr-SEC7-mRFP*
MSY778	*MATα his3Δ1 leu2Δ0 lys2Δ0 ura3Δ0 trs65Δ::KanMX pRS315-GFP-YPT6pRS316-ADH1pr-SEC7-mRFP*
MSY779	*MATα his3Δ1 leu2Δ0 lys2Δ0 ura3Δ0 YEL048cΔ::KanMX pRS315-GFP-YPT6 pRS316-ADH1pr-SEC7-mRFP*
MSY781	*MATα his3Δ1 leu2Δ0 lys2Δ0 ura3Δ0 ric1Δ::KanMX pRS315-GFP-YPT6pRS316-ADH1pr-SEC7-mRFP*
MSY782	*MATα his3Δ1 leu2Δ0 lys2Δ0 ura3Δ0 gyp6Δ::KanMX pRS315-GFP-YPT6pRS316-ADH1pr-SEC7-mRFP*
MSY786	*MATα his3Δ1 leu2Δ0 lys2Δ0 ura3Δ0 pRS315-GFP-YPT6pRS306-ADH1pr-mRuby-SED5*
MSY787	*MATα his3Δ1 leu2Δ0 lys2Δ0 ura3Δ0 trs65Δ::KanMX pRS315-GFP-YPT6pRS306-ADH1pr-mRuby-SED5*
MSY790	*MATα his3Δ1 leu2Δ0 lys2Δ0 ura3Δ0 gyp6Δ::KanMX pRS315-GFP-YPT6pRS306-ADH1pr-mRuby-SED5*
MSY800	*MATα his3Δ1 leu2Δ0 lys2Δ0 ura3Δ0 trs65Δ::KanMX pRS315-GFP-YPT6pRS316-ADH1pr-SEC7-mRFP pRS313*
MSY801	*MATα his3Δ1 leu2Δ0 lys2Δ0 ura3Δ0 trs65Δ::KanMX pRS315-GFP-YPT6pRS316-ADH1pr-SEC7-mRFP pRS313-TRS65*
MSY806	*MATα his3Δ1 leu2Δ0 lys2Δ0 ura3Δ0 trs85Δ::KanMX pRS315-GFP-YPT6pRS316-ADH1pr-SEC7-mRFP*
MSY868	*MATa his3-11 leu2-3,112 ura3-1 ade2-1 trp1Δ can100 trs130Δ50^*ts*^::TRP1 pRS315-GFP-YPT6pRS316-ADH1pr-SEC7-mRFP*
MSY869	*MATa his3-11 leu2-3,112 ura3-1 ade2-1 trp1Δ can100 trs130Δ50^*ts*^::TRP1 pRS315-GFP-YPT31 pRS316-ADH1pr-SEC7-mRFP MATa his3*
MSY876	*ura3-52 lys2 leu2:: LYS2 pRS315-GFP-YPT6pRS316-ADH1pr-SEC7-mRFP*
MSY877	*MATa his3 ura3-52 lys2 leu2:: LYS2 ypt31Δ::HIS3 ypt32A141D pRS315-GFP-YPT6pRS316-ADH1pr-SEC7-mRFP*

### Tandem affinity purification (TAP) and mass spectrometry

Yeast cells were grown to log phase in YPD medium and ~20 g of cells were collected and flash frozen in liquid nitrogen and stored at −80°C. Pellets were resuspended in an equal volume of lysis buffer (6 mM Na_2_H_2_PO_4_/4 mM NaH_2_PO_4_/1% CHAPS/100 mM NaCl/2 mM EDTA/1 mM EGTA/50 mM NaF/0.1 mM Na_3_VO_4_/20 mM β-mercaptoethanol/1 mM PMSF/2 mM benzamidine/leupeptin/pepstatin), lysed by bead beating (10 s on and 10 s off for a total of 10 times) and centrifuged at 21,000 g for 25 min in a JA25.50 rotor. The resulting supernatant was incubated with 300 μL of a 50% slurry of IgG beads for 2 h at 4°C while nutating. The lysate and bead mixture was transferred to a polyprep column and washed three times with 10 mL of wash buffer (10 mM Tris-HCl, pH 8.0/100 mM NaCl/0.1% CHAPS/1 mM DTT), one time with 10 mL of TEV-C buffer (10 mM Tris-HCl, pH 8.0/100 mM NaCl/0.1% CHAPS/0.5 mM EDTA/5% glycerol/1 mM DTT) and one time with 200 μL of TEV-C buffer with 5 μg/mL of recombinant TEV protease. Beads were then incubated with 1mL of TEV-C buffer with 5 μg/mL of recombinant TEV protease for 2 h at 16°C while nutating. The eluate combined with two 1mL TEV-C buffer washes was transferred to a new polyprep column and the following was added: 6 mL of CAM-B buffer (10 mM Tris-HCl pH 8.0/100 mM NaCl/0.1% CHAPS/1 mM MgOAc_2_/1 mM imidazole/2 mM CaCl_2_/5% glycerol/10 mM β-mercaptoethanol), 9 μL of 1 M CaCl_2_, and 250 μL of a 50% slurry of calmodulin beads. The mixture was nutated for 2 h at 4°C and the beads were washed 3 times with 1.5 mL of CAM-B buffer. The beads were incubated with 250 μL of CAM-E buffer (10 mM Tris-HCl pH 8.0/100 mM NaCl/0.1% CHAPS/1 mM MgOAc_2_/1 mM imidazole/10 mM EGTA/5% glycerol) for 5 min and eluate was collected. A total of five fractions were collected, pooled and TCA precipitated. The total amount of precipitated protein was resuspended in sample buffer and loaded onto an SDS-PAGE gel. Peptides were extracted from gel slices either as a total eluate collected at the border between the stacking gel and resolving gel or after fractionation in the resolving gel. Recovered peptides were solubilized (5% acetonitrile, 0.1% formic acid in dH_2_O) and loaded onto a sample column (3 μm PepMap100, 2 cm, 75 μm diameter) using an ultrahigh pressure liquid chromatography system (Easy nLC 1000 system, Thermo-Fisher, San Jose, CA). Bound peptides were then eluted with a 4 h acetonitrile/formic acid continuous gradient at a flow rate of 250 nanoliters per minute into an in-line 50 cm separating column (2 μm PepMap C18, 75 μm diameter) at 40°C. Separated peptides were ionized using an Easy Spray nano source and subjected to MS/MS analysis using a Velos Orbitrap (Thermo-Fisher, San Jose, CA). Precursor scans were performed in the 380–1400 m/z range at 30,000 resolution. Top 6 doubly, triply or quadruply charged ions with intensity higher than 5000 counts were submitted to fragmentation. Optimal accumulation times were set using adaptive automatic gain control with a maximum accumulation time of 150 ms. Precursor ions were selected for fragmentation and re-selected one more time within a 6 s window then put in a dynamic exclusion list of 100. MS/MS scan range was automatically adjusted based on precursor m/z and charge state. Selected ions were fragmented using a normalized fragmentation energy set at 35% and an isolation window of 2 m/z. Data was analyzed using SEAQUEST software.

### Immunoprecipitations

Yeast strains were grown to log phase in YPD medium and approximately 50 OD_600_ units of cells were converted to spheroplasts in spheroplast buffer (1.4 M sorbitol/50 mM KPi/36 mM β–mercaptoethanol/33 μg/ml zymolyase) for 30 min at 37°C. Spheroplasts were washed with wash buffer (1.4 M sorbitol/50 mM KP_i_) and lysed in 1 mL of lysis buffer (20 mM HEPES, pH 7.3/150 mM NaCl/1 mM DTT/2 mM EDTA/1% Triton X-100/1xprotease inhibitor cocktail) and lysates were cleared by centrifugation at 16,000 g for 15 min. Lysates were diluted to 1 mg/mL with lysis buffer and 500 μL was incubated with or without 0.5 μg of anti-HA (Sigma clone 7) antibody for 60 min on ice. Lysates were incubated with 20 μL of a 50% slurry of protein-G beads while nutating at 4°C for 30 min. Beads were washed 2 times with 500 μL of lysis buffer and eluted by boiling in 25 μL of sample buffer for 3 min. Eluted proteins were separated on an SDS-PAGE gel and detected by Western blot analysis.

### Serial dilutions

Yeast strains were inoculated into 3 mL of minimal medium and grown in a shaker incubator overnight at 30°C. The OD_600_ was normalized to the lowest value and 10-fold serial dilutions were spotted (2 μL) onto minimal selective medium. Yeast were placed at the permissive temperature of 30°C to serve as a growth control and at the restrictive temperature of 37°C.

### Microscopy and quantification

Yeast strains were inoculated into 3mL of minimal medium and grown overnight at 30°C to log phase. Cells were pelleted and resuspended in fresh medium to an OD_600_ of 10–15 and visualized on a Nikon Eclipse TiE inverted epifluorescence microscope using a 100 × objective. Sections of 0.5 μm were collected and deconvoluted using AutoQuant X3 software. Co-localization between GFP-Ypt6p and Sec7p-mRFP punctae was manually determined for each cell by counting the number of GFP-Ypt6p punctae that overlapped with Sec7p-mRFP punctae and dividing this amount by the total number of GFP-Ypt6p punctae present. All images were captured under the same microscope settings and the area of GFP-Ypt6p punctae was measured manually using the Freehand selection tool in ImageJ.

### Yeast two hybrid

Genes were cloned into the yeast two hybrid vectors, pGADT7 or pGBKT7, and transformed into AH109 and Y187 yeast strains, respectively. Diploids were selected from matings between AH109 and Y187 strains carrying the appropriate genes on minimal medium not supplemented with leucine or tryptophan. Growth of diploids on medium not supplemented with histidine was used to indicate a positive or negative interaction.

### Subcellular fractionation

Cells were grown to log phase overnight and approximately 50 OD_600_ units of cells were spheroplasted (as above). Spheroplasts were resuspended in lysis buffer (50 mM Tris-HCl, pH7.4/200 mM sorbitol/1mM EDTA/protease inhibitors), homogenized with a Dounce homogenizer (10 strokes) and lysates were centrifuged at 500 g for 5 min. The resulting supernatant was centrifuged at 13,000 g for 10 min to generate a pellet (P13) and supernatant (S13). S13 was then centrifuged at 100,000 g for 60 min with a TLA100 rotor to generate a pellet (P100) and a supernatant (S100). Pellets were resuspended in sample buffer and equivalent amounts of pellets and supernatants were loaded onto an SDS-PAGE gel and analyzed by Western blot.

## Results

### TRAPP II interacts directly with Gyp6

**Figure 1:**
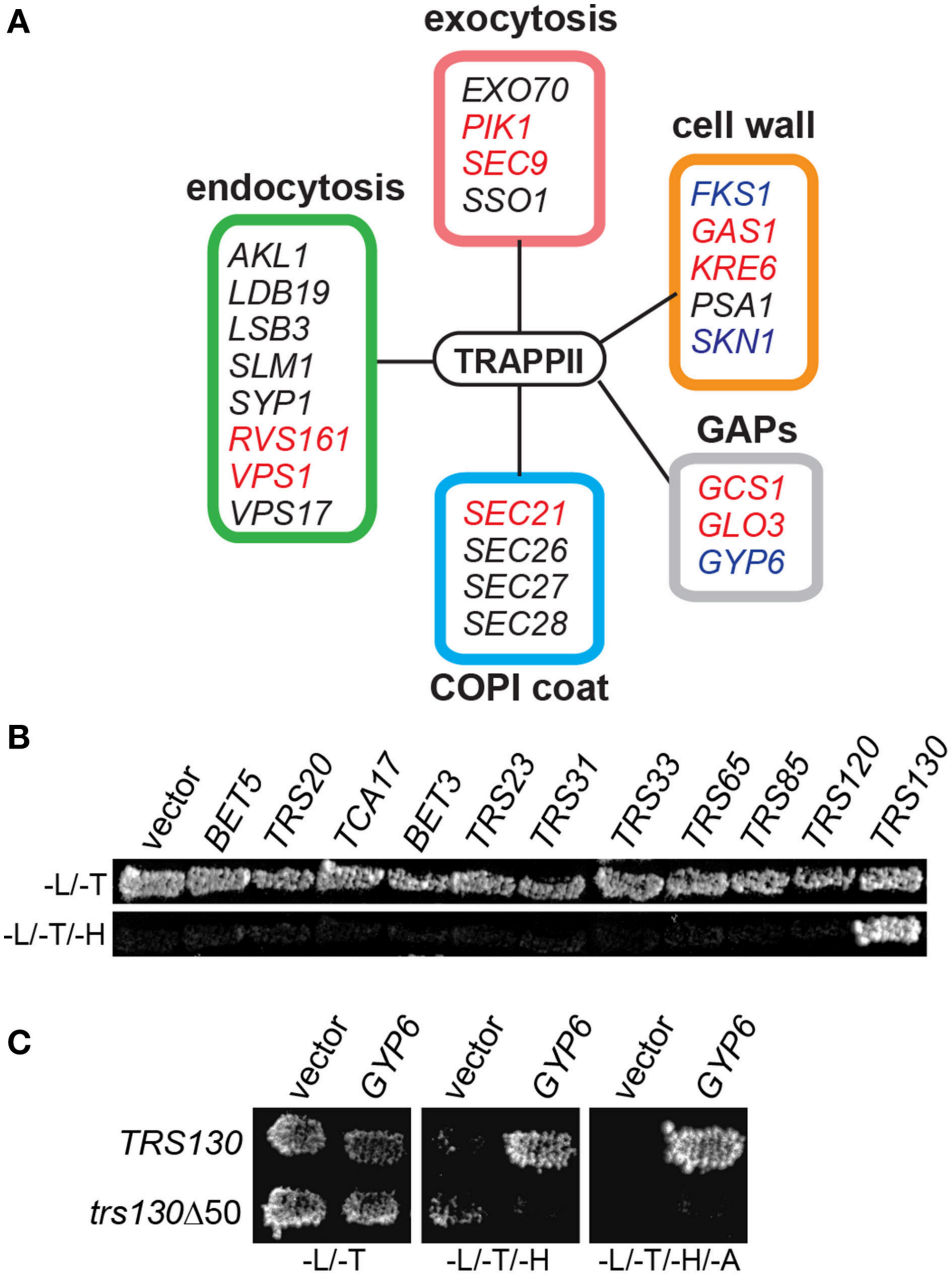
**Gyp6p interacts with the TRAPP II complex through Trs130p. (A)** Trs120p-TAP was purified from cells and co-purifying proteins were identified by mass spectrometry. Peptides from various proteins regulating different membrane trafficking pathways were identified as putative TRAPP II interactors by this method. Genes known to have synthetically lethal interactions with mutations in TRAPP II subunit genes are indicated in red. Proteins reported to physically interact with subunits of the TRAPP II complex are indicated in blue. The box coloring around the groups of genes corresponds to their coloring in Table 3. **(B)** In a yeast two hybrid assay, Trs130p (fused to the DNA binding domain of Gal4p) is the only TRAPP subunit showing a positive interaction with Gyp6p (fused to the activation domain of Gal4p) indicated by growth on a plate lacking histidine. -L/-T are plates lacking leucine and tryptophan; -L/-T/-H are plates lacking leucine, tryptophan and histidine. **(C)** The interaction between Gyp6 and Trs130p was lost when the carboxy-terminal 50 amino acids of Trs130p were deleted. -L/-T are plates lacking leucine and tryptophan; -L/-T/-H are plates lacking leucine, tryptophan and histidine; -L/-T/-H/-A are plates lacking leucine, tryptophan, histidine and adenine.

TRAPP II is a large complex with 10 unique subunits that likely interacts with a variety of proteins. To identify with which factors the TRAPP II complex interacts, native yeast TRAPP II was purified from a haploid yeast strain whose endogenous copy of Trs120p was fused to a Tandem Affinity Purification (TAP) tag at the carboxy terminus. Co-purifying proteins were identified either by excising unique bands from an SDS-polyacrylamide gel or by analyzing the total eluate (Figure 1A and Table 3). All 10 TRAPP II subunits were detected and the TRAPP III-specific protein, Trs85p, was not, showing the specificity of the technique. Although purported to be a GEF for Ypt1p and the related GTPases Ypt31p and Ypt32p, none of these three GTPases were detected in our pulldowns. This is not unexpected since the interaction between GEFs and GTPases is transient in nature. A number of proteins involved in TRAPP II-associated pathways including cell wall synthesis, endocytosis and exocytosis were also detected (Figure 1A and Table 3). Several of these proteins have been shown to interact either physically or genetically with TRAPP II subunits. Notably, four of the seven COPI coat subunits co-purified with TRAPP II, in support of previous studies showing an association between TRAPP II and COPI vesicles (Cai et al., 2005; Chen et al., 2011). Interestingly, three GAP proteins functioning in membrane trafficking pathways also co-purified with TRAPP II: the Arf-GAPs Glo3p and Gcs1p, and the Ypt6p Rab-GAP, Gyp6p.

**Table 3:** **Summary of a subset of TRAPP II interactors by tandem affinity purification and mass spectrometry**.

		**Peptides**	
**Accession#**	**Common name**	**Total**	**Unique**
YDR407C	TRS120	131	113
YMR218C	TRS130	93	72
YGR166W	TRS65	39	39
YDR472W	*TRS31*	31	14
YNL287W	SEC21	28	25
YGL137W	SEC27	24	21
YDR238C	SEC26	23	18
YLR342W	*FKS1*	19	16
YDL055C	*PSA1*	18	13
YCR009C	RVS161	18	12
YCR030C	SYP1	18	16
YOR115C	TRS33	18	16
YKR068C	BET3	16	12
YDR246W	TRS23	15	14
YMR307W	GAS1	14	7
YBR059C	AKL1	14	5
YJL044c	GYP6	14	10
YPL232W	SSO1	10	5
YFR024C-A	LSB3	10	8
YGR143W	SKN1	9	2
YER122C	GL03	8	5
YPR159W	KRE6	8	4
YDL226C	GCS1	8	5
YNL267W	PIK1	7	2
YIL105C	SLM1	6	4
YIL076W	SEC28	6	6
YML077W	BET5	6	6
YGR009C	SEC9	5	3
YBR102C	EXO89	5	3
YBR254C	TRS20	5	5
YKR001C	VPS1	4	2
YEL048C	TCA17	4	4
YOR322C	LDB19	3	1
YOR132W	VPS17	3	1

*Selected genes identified by mass spectrometry are listed in order of greatest number of peptides recovered to smallest, and color coded according to their classification in Figure 1A (green, endocytosis; red, exocytosis; orange, cell wall; gray, GAPs; blue, COPI coat). TRAPP subunits recovered from the mass spectrometry analysis are colored in white*.

Proteins that co-purified with TRAPP II are likely a combination of proteins interacting directly and indirectly with the complex. To determine whether Gyp6p directly interacts with the complex, a yeast two-hybrid experiment between full length Gyp6p and all TRAPP II subunits was conducted. As shown in Figure 1B, Gyp6p interacted solely with Trs130p. This interaction was dependent upon the extreme carboxy-terminus of Trs130p since the interaction was lost upon deletion of the carboxy-terminal 50 amino acids (Figure 1C). Separation of Gyp6p into its catalytic and non-catalytic portions (residues 1-309 and 310-458, respectively) abrogated the interaction with Trs130p (not shown) suggesting either that intact Gyp6p is required for the interaction or that the fragments of Gyp6p are unstable. Collectively, these results suggest that Gyp6p interacts directly with the TRAPP II complex via the carboxy-terminal portion of the TRAPP II-specific subunit, Trs130p.

### *YPT6* and its GEF interact genetically with TRAPP II and TRAPP III subunits

**Figure 2:**
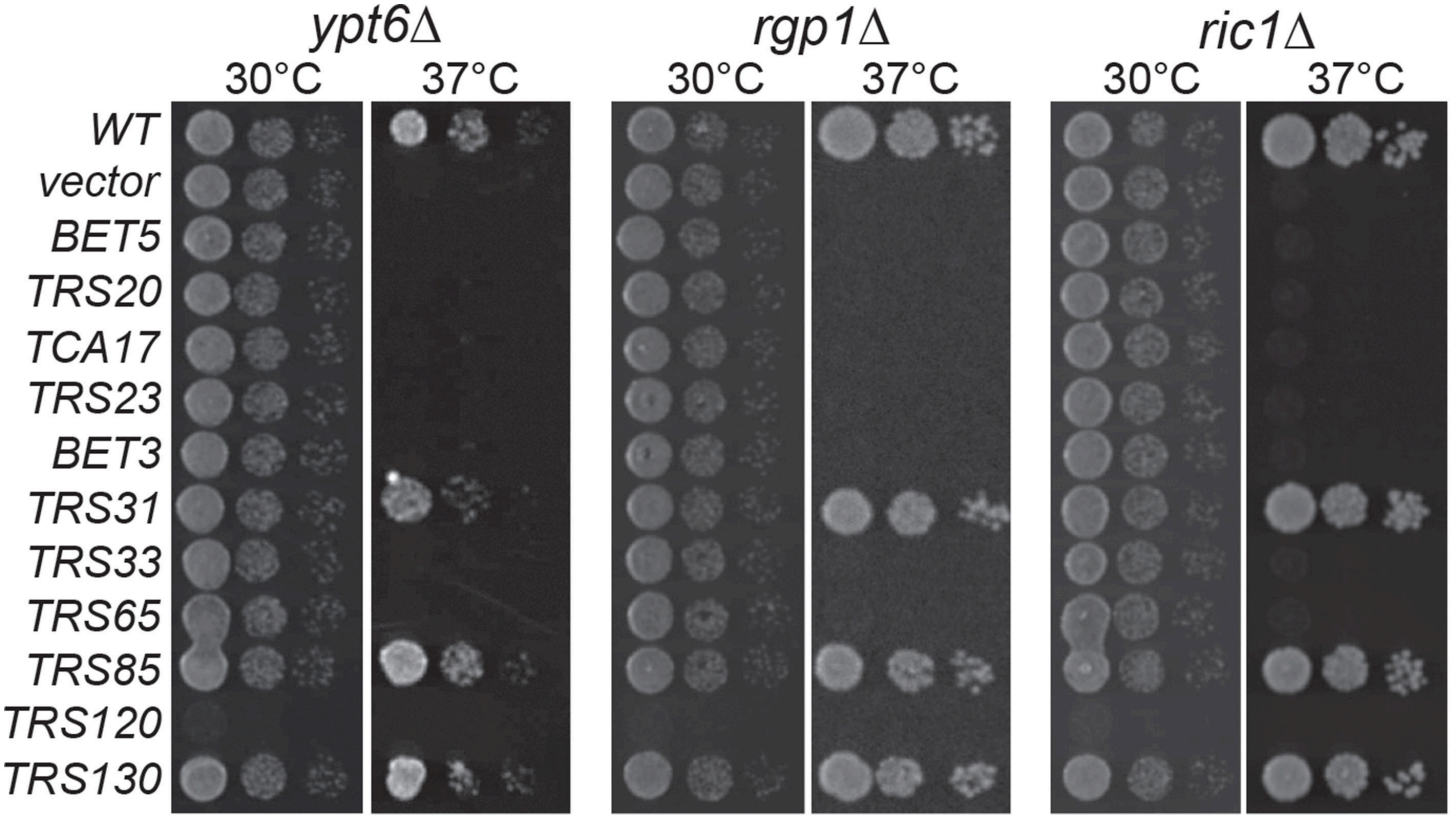
**TRAPP II and III subunits genetically interact with *YPT6* pathway genes**. TRAPP subunits were overexpressed in *ypt6*Δ, *rgp1*Δ and *ric1*Δ strain backgrounds and serial dilutions were plated and grown at permissive (30°C) and restrictive (37°C) temperatures. WT indicates the wild type gene for each respective strain and vector indicates an empty vector.

In high throughput screens, several genetic interactions have been documented between *YPT6* pathway genes and TRAPP II subunits (Tong et al., 2004; Costanzo et al., 2010; Hoppins et al., 2011). To examine how *YPT6* pathway genes genetically interact with essential and non-essential TRAPP II subunits, a high copy suppression analysis was conducted (Figure 2). Included in this study were *RIC1* and *RGP1*, genes that encode the subunits of the heterodimeric Ypt6p GEF. Genes encoding TRAPP subunits expressed in high copy plasmids were transformed into *ypt6*Δ, *ric1*Δ and *rgp1*Δ mutants that are unable to grow at 37°C. The core TRAPP subunit gene *TRS31* suppressed temperature sensitivity in all three mutants although the phenotypic suppression was slightly better in *ric1*Δ and *rgp1*Δ mutants when compared to *ypt6*Δ. This might suggest that the requirement for active Ypt6p is being bypassed. Previous studies have reported that overexpression of *YPT1* permits the growth of *ypt6*Δ at restrictive temperatures and rescues trafficking defects associated with this mutant (Li and Warner, 1998; Ye et al., 2014). Thus, the suppression of *ric1*Δ and *rgp1*Δ seen with *TRS31* overexpression may be indirect and mediated through *YPT1*. Interestingly, overexpression of the TRAPP II-specific essential gene *TRS120* made all three strains very sick even at permissive temperature. This effect is specific to the *YPT6* pathway, since overexpression of *TRS120* had no effect on a wild type strain (data not shown) and the same construct was able to suppress other mutant phenotypes (Scrivens et al., 2009). Redundant pathways likely exist to compensate for the absence of Ypt6p and one possibility is that Trs120p interacts with proteins required for these redundant pathways and sequesters them when it is overexpressed (Li and Warner, 1998; Benjamin et al., 2011). Overexpression of *TRS130*, the gene encoding a second essential TRAPP II-specific subunit, was able to efficiently suppress the growth defect of all three strains. Curiously, overexpression of the gene *TRS85* that encodes a TRAPP III-specific subunit was also efficiently capable of suppressing the growth defect of all three strains. These suppression analyses demonstrate that Gyp6p genetically interacts with both TRAPP II and TRAPP III.

### The integrity of TRAPP II is important for interacting with Gyp6

**Figure 3:**
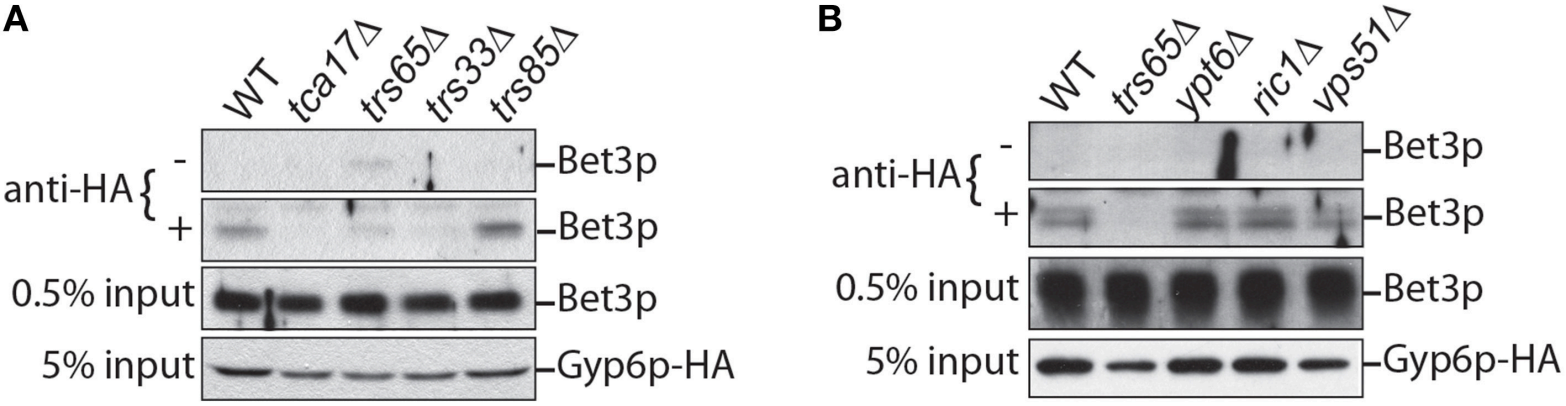
**TRAPP II co-precipitates with endogenous Gyp6p-HA. (A)** A small amount of TRAPP II (Bet3p is used as a marker) co-precipitates with Gyp6p-HA immunoprecipitated from yeast lysates. This interaction is disrupted in mutants (*tca17*Δ, *trs65*Δ and *trs33*Δ) that affect assembly of the TRAPP II but not the TRAPP III (*trs85*Δ) complex. **(B)** TRAPP II continues to interact with Gyp6p-HA when non-essential components of the Ypt6p pathway are absent. Co-immunoprecipitations were performed in strain backgrounds harboring deletions of either *YPT6, RIC1* or *VPS51*, a Ypt6p-effector.

In order to continue characterizing the interaction between Gyp6p and TRAPP II, co-immunoprecipitation experiments were conducted in wild type and mutant yeast strains. Of the ten TRAPP II subunits, Trs33p, Tca17p, and Trs65p are non-essential and gel filtration experiments have shown that these subunits are important for complex assembly (Tokarev et al., 2009; Choi et al., 2011). To examine whether the assembly of the TRAPP II complex is important for its interaction with Gyp6p, the endogenous copy of *GYP6* was tagged at the carboxy terminus with a triple hemagglutinin (HA) tag and immunoprecipitated from cell lysates. Bet3p, a core TRAPP subunit, was detected by western analysis to assay for the presence of TRAPP II (Figure 3A). In wild type cells, TRAPP II co-precipitated with Gyp6p-HA, confirming Gyp6p as an interacting partner of the TRAPP II complex. This interaction was abrogated in lysates from *trs65*Δ, *tca17*Δ, and *trs33*Δ mutants, indicating that proper assembly of the TRAPP II complex is important for its association with Gyp6p (Figure 3A). The deletion of *TRS33* and *TCA17* have been shown to destabilize the TRAPP II complex, while deletion of *TRS65* interferes with TRAPP II dimerization as intact TRAPP II monomers can be purified from *trs65*Δ cells (Yip et al., 2010). It is surprising that Gyp6p would not be able to associate with TRAPP II monomers which contain one copy of Trs130p. It is possible that disrupting TRAPP II dimerization indirectly affects its association with Gyp6p. For example, dimerization may be important for TRAPP II localization to the site of Gyp6p interaction, or it may be required for upstream events. Deleting the TRAPP III-specific subunit *TRS85* had no effect on the amount of Bet3p that co-precipitated with Gyp6p-HA (Figure 3A) suggesting that the TRAPP III complex does not alter the interaction of Gyp6p with TRAPP II. A small percentage of Bet3p (<0.05%) co-precipitated with Gyp6p-HA despite a very efficient pull-down (not shown), suggesting that the interaction between TRAPP II and Gyp6p is weak and likely transient.

Genetic interactions between TRAPP II and *YPT6* pathway genes suggest that the interaction between TRAPP II and Gyp6p may be important for regulating Ypt6p pathways. Gyp6p is a GAP for Ypt6p and to determine whether active Ypt6p must be present for TRAPP II to interact with Gyp6p, the same co-immunoprecipitation experiment was conducted in *ypt6*Δ, *ric1*Δ, and *rgp1*Δ mutant strains. TRAPP II and Gyp6p interacted to the same degree in these mutants as wild type (Figure 3B), indicating that Ypt6p, active or not, is not required for TRAPP II and Gyp6p to interact. A possible interpretation of this result is that Gyp6p interacts with TRAPP II upstream of its association with Ypt6p.

### Ypt6p is enriched at the late golgi in a *trs65*Δ mutant

**Figure 4:**
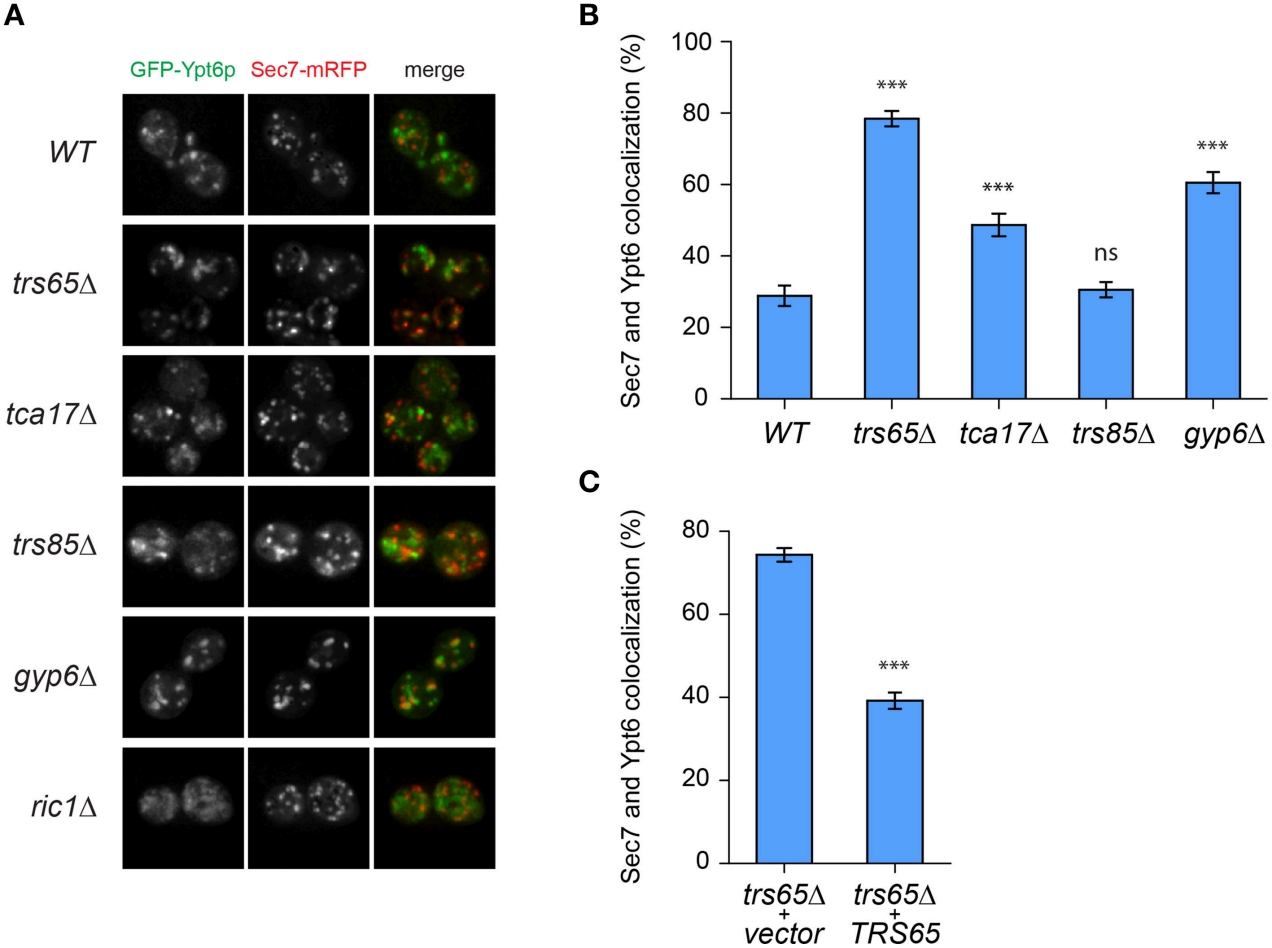
**GFP-Ypt6p becomes enriched at the late Golgi in a *trs65*Δ mutant. (A)** The localization of GFP-Ypt6p was examined in different strain backgrounds and compared to Sec7-mRFP localization, a late Golgi marker. A representative field of 1–2 cells is shown for each strain background. **(B)** The percentage of GFP-Ypt6p punctae co-localizing with Sec7p-mRFP was quantified in each strain. Significance was assessed between wild type and each mutant using a one way ANOVA. *Post-hoc* differences were made using Fisher's probability of least squared differences. *P*-values are indicated by asterisks (^***^ ≤ 0.001). **(C)** Co-localization of GFP-Ypt6p and Sec7p-RFP was quantified in a *trs65*Δ strain transformed with either empty vector (*vector*) or wild type *TRS65*. Significance was assessed as indicated in **(B)**. For **(B,C)**, 56–96 cells, representing 10–15 fields with 3–10 cells each were counted for each strain. Error bars represent standard error of the mean (SEM). Significance in **(B)** was assessed using an unpaired *t*-test. *P*-values are indicated by asterisks (^***^ ≤ 0.001).

Recent work showed that Gyp6p is a putative effector of Ypt32p and in a *gyp6*Δ mutant, Ypt6p remains at the late Golgi for a longer time and has increased co-localization with Ypt32p (Suda et al., 2013). We speculated that TRAPP II is involved in a similar mechanism, and may also recruit Gyp6p to the late Golgi to promote Ypt6p inactivation and dissociation from this organelle. Wild type and mutant strains were transformed with two plasmids, one expressing GFP-Ypt6p (Ypt6p) and another expressing Sec7p-mRFP (Sec7p), a marker for the late Golgi. Ypt6p and Sec7p displayed a punctate localization pattern, shown in representative fields for each strain (Figure 4A). The percentage of co-localized punctae was quantified and in wild type cells, 29% of Ypt6p co-localized with Sec7p (Figure 4B), which is consistent with a previous study (Kawamura et al., 2014). Co-localization increased almost 3-fold to 79% in a *trs65*Δ mutant (Figure 4B). A less dramatic increase in co-localization was observed in the TRAPP II mutant *tca17*Δ (49%) and in a *gyp6*Δ mutant (61%) (Figure 4B). The absence of Trs85p, a TRAPP III-specific subunit, had no effect on co-localization (31%) (Figure 4B).

To confirm that the dramatic increase in localization of Ypt6p to the late Golgi observed in *trs65*Δ is caused by the deletion of *TRS65* we attempted to rescue this phenotype. A plasmid expressing the wild type copy of *TRS65* was transformed into the *trs65*Δ strain and co-localization between Ypt6p and Sec7p was quantified. When wild type *TRS65* was introduced into *trs65*Δ cells, the level of co-localization approached wild type levels (39%; Figure 4C) suggesting that increased co-localization between Ypt6p and Sec7p was caused by the deletion of *TRS65*.

**Figure 5:**
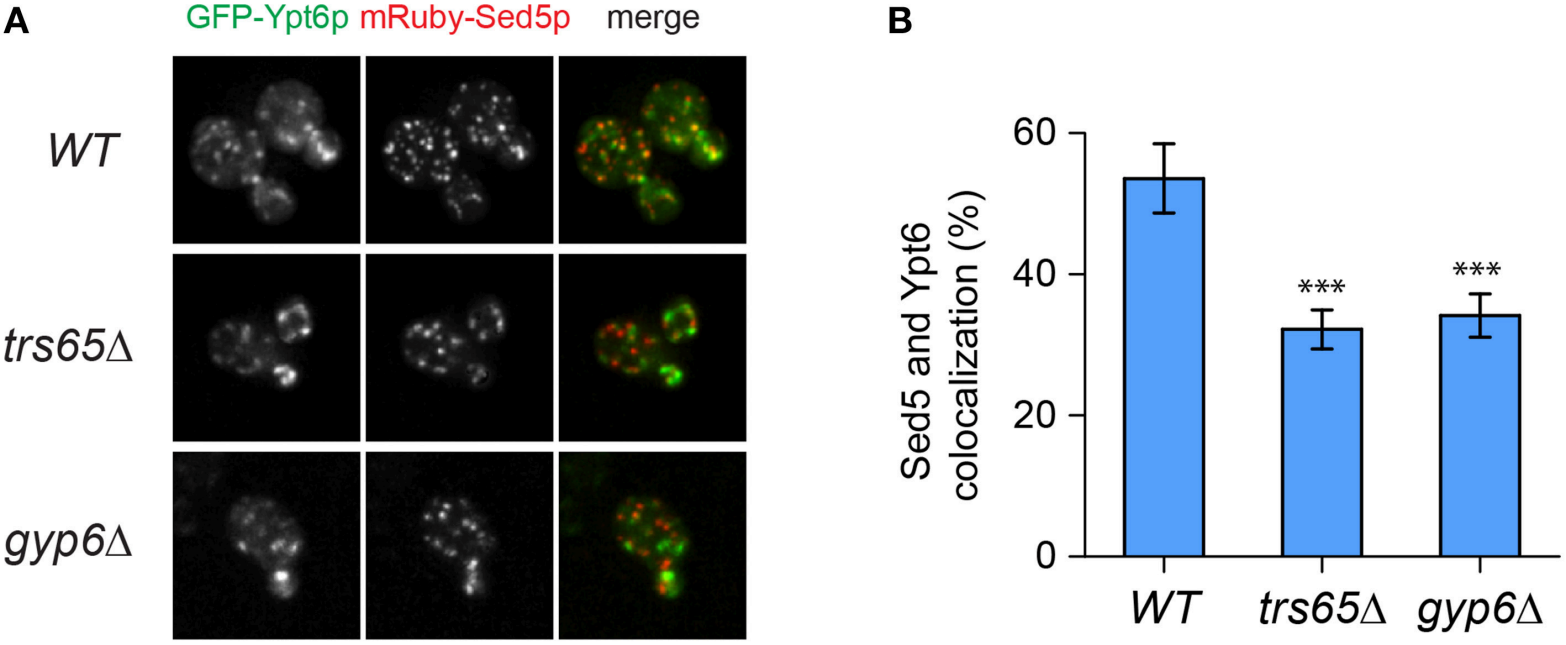
**Co-localization of GFP-Ypt6p with the early Golgi is decreased in a *trs65*Δ and *gyp6*Δ mutant. (A)** The localization of GFP-Ypt6p was examined in wild type (WT), *trs65*Δ and *gyp6*Δ cells and compared to mRuby-Sed5p localization, an early Golgi marker. A representative field of 1–2 cells is shown for each strain. **(B)** The percentage of GFP-Ypt6p punctae co-localizing with mRuby-Sed5p was quantified in each strain. Significance was assessed as described in the legend to Figure 4. *P*-values are indicated by asterisks (^***^ < 0.001). 31–38 cells representing 5–6 fields of 3–10 cells were counted for each strain. Error bars represent SEM.

Ypt6p localizes to both the early and late Golgi, making it plausible that a portion of the punctae not co-localizing with Sec7p represent early Golgi structures (Suda et al., 2013; Kawamura et al., 2014). To verify this, co-localization between Ypt6p and the early Golgi marker Sed5p was examined (Figure 5A). The opposite trend as Sec7p was observed as Ypt6p co-localized with Sed5p more often in wild type (54%) than *trs65*Δ (32%) and *gyp6*Δ (34%) cells (Figure 5B). These results, together with the co-localization data for Sec7p, suggest that the punctate localization of Ypt6p is caused by its association with Golgi compartments, and de-stabilization of TRAPP II oligomers by the deletion of *TRS65* causes an enrichment of Ypt6p at the late Golgi.

### The localization pattern of GFP-Ypt6p varies between wild type and mutant strains

**Figure 6:**
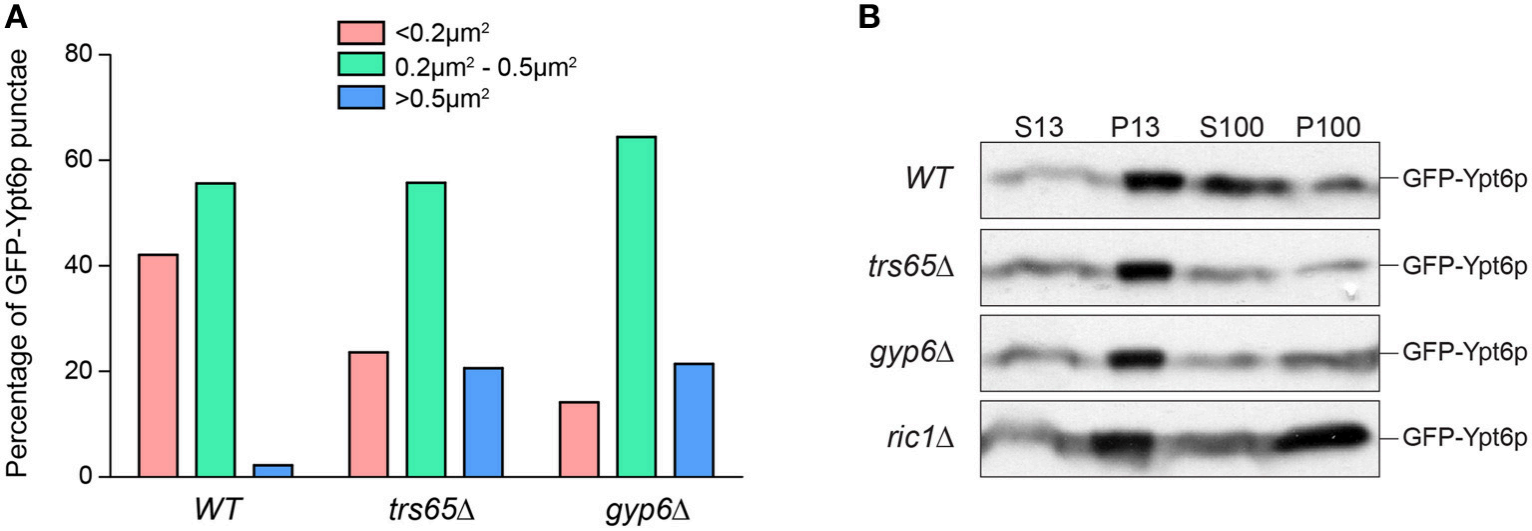
**Higher frequencies of large punctae are seen in *trs65*Δ and *gyp6*Δ mutants. (A)** The area of GFP-Ypt6p punctae was measured with ImageJ, divided into three categories (< 0.2 μm^2^, 0.2–0.5 μm^2^ or >0.5 μm^2^) and the frequency of each category was plotted for wild type (WT), *trs65*Δ and *gyp6*Δ strains. Between 107 and 154 punctae were counted for two experiments. **(B)** Subcellular membrane fractionation was performed in WT and mutant strains and GFP-Ypt6p was probed for in each fraction.

In addition to differences in co-localization with Sec7p, Ypt6p punctae in *trs65*Δ and *gyp6*Δ mutants appear larger and more prominent when compared to wild type. Large variation was seen in the measured area for punctae but interestingly, 21% of punctae in both *trs65*Δ and *gyp6*Δ were larger than 0.5 μm^2^, whereas only 2% of wild type punctae met this criterion (Figure 6A). Taken together with the increased co-localization between GFP-Ypt6 and Sec7-RFP (Figures 4A,B), these larger punctae likely represent an accumulation of Ypt6p on late Golgi membranes.

Another more striking difference in the distribution of Ypt6p can be observed in *ric1*Δ cells, as GFP-Ypt6p is not punctate but is diffusely localized throughout the cell (Figure 4A). The same phenotype was seen in *rgp1*Δ cells (data no shown). This diffuse localization suggests that Ypt6p is cytosolic but membrane fractionation showed no increase in the ratio of cytosolic to membrane-bound Ypt6p relative to wild type (Figure 6B). In the absence of its GEF, Ypt6p does not associate with punctate Golgi structures but does remain associated with membranes. The original study characterizing Ric1p/Rgp1p also observed this localization and hypothesized that inactive Ypt6p localizes to smaller membrane structures such as vesicles (Siniossoglou et al., 2000). Alternatively, partial mislocalization of Ypt6p to the ER has been reported in the absence of its GEF (Cabrera and Ungermann, 2013) suggesting that membrane-bound Ypt6p may be associated with the ER in the *ric1*Δ mutant.

### The effect of TRAPP II on Gyp6P is not entirely mediated by Ypt31p and Ypt32p

**Figure 7:**
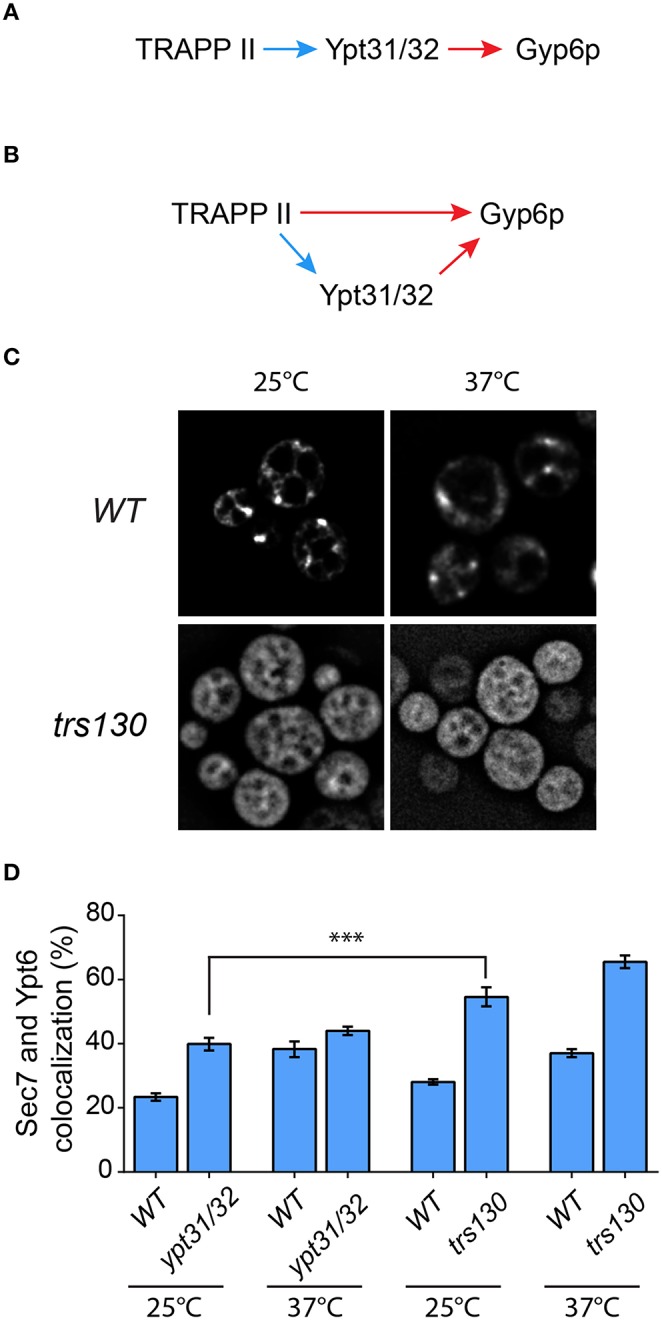
**The TRAPP II-Gyp6p interaction is not mediated by Ypt31/32**. Two models for the recruitment of Gyp6p to the Golgi are presented. In **(A)**, TRAPP II activates Ypt31/32 via its purported GEF activity toward these GTPases. The activated GTPases then interact with the effector Gyp6p. Alternatively **(B)**, both TRAPP II and Ypt31/32 interact with Gyp6p. The Ypt31/32-Gyp6p interaction is preceded by activation of Ypt31/32 by TRAPP II GEF activity, whereas TRAPP II interacts with Gyp6 through the Trs130p subunit. Blue arrows indicate GEF activity while red arrows indicate a physical interaction. **(C)** The localization of GFP-Ypt31p was examined in *trs130*. A representative field is shown for the mutant and its isogenic wildtype strain at 25 and 37°C. **(D)** The percentage of GFP-Ypt6p punctae co-localizing with Sec7-mRFP was quantified in each of the indicated mutants and their respective isogenic wildtype strains. 41–50 cells representing 5–6 fields of 3–10 cells were counted for each strain. Error bars represent SEM. Significance was assessed as described in the legend to Figure 4. *P*-values are indicated by asterisks (^***^ < 0.001).

A recent study suggested that Gyp6p is an effector of the related GTPases Ypt31p and Ypt32p (Suda et al., 2013). Although disputed (Wang and Ferro-Novick, 2002; Cai et al., 2008), TRAPP II has been suggested to be the GEF for these GTPases (Jones et al., 2000; Morozova et al., 2006) and a recent study has strengthened this notion (Kim et al., 2016a). Therefore, our data above could be explained by a simple model (Figure 7A) in which TRAPP II activates Ypt31/32 which then recruits Gyp6p, thus regulating Ypt6p localization and dynamics at the Golgi. Alternatively, our data could also support a model whereby Ypt6p localization is regulated directly by both TRAPP II and Ypt31/32 (Figure 7B).

In order to distinguish between these two models we first examined the localization of Ypt31p and Ypt6p in a *trs130* yeast mutant. This mutant has a truncation of the final 50 amino acids of Trs130p rendering it heat sensitive (Sacher et al., 2001). As shown in Figure 7C (also see Morozova et al., 2006; Cabrera and Ungermann, 2013), the localization of GFP-Ypt31 was altered in *trs130* at both the permissive (25°C) and the restrictive (37°C) temperatures. At 25°C the punctate Golgi pattern seen in wild type was lost in *trs130* and instead an ER-like pattern was seen. Although some cells retained this ER-like pattern at 37°C, many cells displayed a diffuse pattern of fluorescence. ER-localization of other GTPases has been reported when their respective GEFs are inactivated (Cabrera and Ungermann, 2013), further supporting the notion that TRAPP II is the GEF for Ypt31/32. Loss of the Golgi staining pattern for Ypt31p has been reported for other TRAPP II subunit mutations (Montpetit and Conibear, 2009). Given this result we then measured the co-localization of GFP-Ypt6 with Sec7-mRFP in this mutant. As shown in Figure 7D, at 25°C co-localization was 58% in *trs130* compared to 28% in wild type. Increasing the temperature to 37°C resulted in a small increase in co-localization in *trs130* (65%) but a more dramatic increase was seen in wild type (37%). The reason for the increase in wild type at this elevated temperature is not known.

We then examined a mutant (*ypt31/32*) in which *YPT31* has been deleted and *YPT32* was replaced with a heat sensitive A141D mutation (Jedd et al., 1997). In the *ypt31/32* mutant, there was a small increase in the co-localization between GFP-Ypt6 and Sec7-mRFP relative to wild type (38% in *ypt31/32*, 23% for wild type) at 25°C (Figure 7D). The difference in co-localization between *ypt31/32* and *trs130* (see above) was statistically significant, with a higher value seen for *trs130*. Upon temperature shift, as seen for the *trs130* isogenic wild type, there was a large increase in co-localization between these proteins in the *ypt31/32* isogenic wild type strain (39%) but only a small increase was seen in *ypt31/32* (43%) (Figure 7D). Thus, we conclude that in the absence of functional Ypt31p and Ypt32p the Gyp6 protein is still partially recruited to the Golgi. Overall, the defect in *ypt31/32* at 25°C was not as dramatic as that seen for any of the TRAPP II subunit mutations examined including *tca17, trs65*, and *trs130*. Taken together with the lack of detection of both Ypt31p and Ypt32p in the Trs120-TAP purification that identified Gyp6p (Figure 1A), our data support the model (Figure 7B) whereby both TRAPP II and Ypt31/32 contribute to the recruitment of Gyp6p to the Golgi in order to regulate Ypt6p dynamics at this organelle.

## Discussion

Ypt6p is a non-essential Rab protein that has been implicated in several trafficking pathways in both yeast and higher eukaryotes. This study has shown that the TRAPP II complex interacts with the Ypt6p GAP Gyp6p, and when this interaction is disrupted *in vivo*, Ypt6p becomes enriched on late Golgi compartments. This physical interaction is supported by two independent high-throughput studies employing pull-downs coupled to mass spectrometric analysis that each demonstrated an interaction between Gyp6p and TRAPP subunits (Ho et al., 2002; Krogan et al., 2006). Several studies have examined the subcellular localization of Ypt6p by microscopy and have found that it localizes to all three (early/medial/late) Golgi compartments, COPI vesicles and late endosomes (Suda et al., 2013; Kawamura et al., 2014). The level of early Golgi localization and late Golgi localization reported varies from ~50–80% and ~30–50%, respectively. The results in this study fall within this range: in wild type cells Ypt6p punctae localized to the early and late Golgi with a frequency of 54 and 29%, respectively. Ypt6p appears to reside in more than one subcellular compartment, consistent with it regulating several trafficking pathways.

Gyp6p recruitment to different organelles by various protein factors could be a mechanism to promote the exit of Ypt6p from a specific compartment and maintain its homeostatic distribution throughout the cell. In this case, preventing the recruitment of Gyp6p would skew the localization of Ypt6p toward one compartment at the expense of another. This does indeed occur in the TRAPP II mutant *trs65*Δ, as Ypt6p co-localization with the early Golgi is reduced, while it is enriched more than 2-fold at the late Golgi. A similar pattern of re-distribution was observed in a *gyp6*Δ mutant, suggesting that the change in Ypt6p localization in *trs65*Δ is the result of TRAPP II no longer efficiently recruiting Gyp6p to the late Golgi.

The inactivation of Rabs is associated with a non-membrane-bound, cytosolic state, but in the absence of its GEF, the ratio of membrane bound to cytosolic Ypt6p does not change. However, there is a striking difference in Ypt6p localization in *ric1*Δ and *rgp1*Δ mutants compared to wild type. It was recently shown that Ypt6p, Ypt7p and Vps21p re-localize to the ER in the absence of their GEFs, suggesting that GEFs are required for localization to the correct organelle, and not necessarily required for association with membranes (Cabrera and Ungermann, 2013). It is likely that several factors coordinate the spatial and temporal localization of Ypt6p by ensuring that its modifying proteins are also correctly localized.

In addition to the TRAPP II complex, Ypt32p has also been shown to interact with Gyp6p and recruit it to the late Golgi, and the Na^+^/H^+^ exchanger Nhx1p interacts with Gyp6p on late endosomes (Ali et al., 2004; Suda et al., 2013). In both cases, this association was proposed to be important for the regulation of Ypt6p. It is interesting that both TRAPP II and Ypt32p interact with Gyp6p, as TRAPP II is a putative Ypt32p GEF and has been shown to act upstream of the Rab in autophagy and post Golgi trafficking pathways (Sciorra et al., 2005; Tokarev et al., 2009; Zou et al., 2013). Ypt6p, TRAPP II, and Ypt31/32 have all been shown to be important for trafficking of the exocytic SNARE Snc1p through the endocytic pathway (Lafourcade et al., 2003; Cai et al., 2005; Chen and Tokarev, 2005). Interestingly, the overexpression of *TRS120* significantly reduced the viability of *ric1*Δ, *rgp1*Δ and *ypt6*Δ cells (this study). Trs120p is important for the function of TRAPP II in endocytic recycling suggesting that both TRAPP II and Ypt32p may interact with Gyp6p to regulate this pathway (Cai et al., 2005; Montpetit and Conibear, 2009). A Rab GAP cascade has already been proposed between Ypt32p and Gyp6p to regulate a not yet specified membrane trafficking pathway (Suda et al., 2013). The double recruitment of Gyp6p by TRAPP II and Ypt32p could act as a positive feedback loop, whereby TRAPP II acts to both recruit Gyp6p directly and indirectly through the activation of Ypt32p. It also remains possible that TRAPP II and Ypt32p interact with Gyp6p to regulate Ypt6p in distinct trafficking pathways that intersect with the late Golgi compartment.

A previous study suggested that Gyp6p is an effector of Ypt31/32 (Suda et al., 2013). If TRAPP II is in fact a GEF for Ypt31/32, a simple interpretation of our data would be as shown in the model in Figure 7A; activation of these GTPases by TRAPP II ultimately leads to the recruitment of Gyp6p to the late Golgi. However, there are several reasons to favor the alternate model shown in Figure 7B. First, while mass spectrometric analysis identified all TRAPP II subunits and a number of unique peptides from Gyp6p, no peptides for Ypt31p and Ypt32p were detected in the purification (Figure 1A). This suggests that the TRAPP II-Gyp6p interaction is not mediated by either of these two GTPases. Further supporting this notion is the fact that the only physical interaction between TRAPP II and Ypt31/32 annotated in the Saccharomyces genome database (http://www.yeastgenome.org) is via a yeast two hybrid assay and not through a pulldown. Second, we demonstrated that a small deletion from the carboxy-terminus of Trs130p was sufficient to disrupt its interaction with Gyp6p by a yeast two-hybrid assay (Figure 1C). Although it is formally possible that this interaction is bridged by another protein, we are unaware of any cases where this has been proven. Finally, although the *ypt31/32* mutant showed an increase in co-localization between GFP-Ypt6 and Sec7-mRFP, this increase was not as high as that seen for any of the TRAPP II mutations examined. It is unlikely that this is due to a leaky mutant since previous studies have documented this mutant, as well as analogous GTPase mutations, as rapid and tight (Moya et al., 1993; Jedd et al., 1995, 1997). Further support for this model comes from the fact that a *ypt32*Δ mutant did not alter Ypt6p-Sec7p co-localization (Suda et al., 2013). Although it was suggested that Ypt31p compensated for the disrupted Ypt32p, the former GTPase did not bind to Gyp6p in the same assay that demonstrated its interaction with Ypt32p. We suggest that the TRAPP II-Gyp6p interaction can account for the recruitment of Gyp6p to late Golgi membranes in the absence of Ypt32p. Future work should help distinguish between the two possible models.

## Author contributions

MS conceived the experiments, analyzed the data and edited the manuscript. SB, DS, MM, and TN performed all the experiments, wrote and edited the manuscript, and analyzed the data.

### Conflict of interest statement

The authors declare that the research was conducted in the absence of any commercial or financial relationships that could be construed as a potential conflict of interest.
